# Abdominal abscess caused by Raoultella ornithinolytica secondary to postoperative gastric fistula: case report and review of literature

**DOI:** 10.1186/s12879-024-09234-y

**Published:** 2024-03-29

**Authors:** Qiuxia Huang, Jihong Zhang, Gang Liao

**Affiliations:** The Central Hospital of Shaoyang, Shaoyang, Hunan China

**Keywords:** Raoultella ornithinolytica, Choledocholithotomy, Postoperative gastric fistula, Non-viscerally abdominal abscess

## Abstract

**Background:**

In recent years, Raoultella ornithinolytica (R. ornithinolytica) have attracted clinical attention as a new type of pathogen. A wide range of infections with these germs is reported, and commonly found in urinary tract infections, respiratory infections, and bacteremia.

**Case presentation:**

We report the case of an elderly woman with liver abscess, choledocholithiasis and cholangitis, who developed gastric fistula and abdominal abscess after underwent choledocholithotomy, and R. ornithinolytica were isolated from the abdominal drainage fluid. The patient was treated with meropenem and levofloxacin and had a good outcome.

**Conclusions:**

To the best of our knowledge, case of isolating R. ornithinolytica from a patient with non-viscerally abdominal abscess was extremely rare. We share a case of a woman with non-viscerally abdominal abscess secondary to postoperative gastric fistula, R. ornithinolytica was isolated from the patient’s pus, and the pathogenic bacteria may originate from the gastrointestinal tract. Based on this case, We should be cautious that invasive treatment may greatly increase the probability of infection with this pathogenic bacterium.

## Background

R. ornithinolytica is an encapsulated, Gram-negative, nonmotile rod belonging to the Enterobacteriaceae family [1]. It is closely related to Klebsiella spp. and easily misidentified as Klebsiella pneumonia or Klebsiella oxytoca. In 2001, with phylogenetic testing including 16_S_ rRNA and rpoB sequence analysis, the bacterium was reclassified as Raoultella. R. ornithinolytica has been found in water environments, soil, insects, fish, ticks, and termites, as well as hospital environments, and is colonized in the digestive tract and upper respiratory tract in the human body. In 2009, Morais and Vos et al. reported the first cases of human infections of R. ornithinolytica [2]. Subsequently, biliary tract infection, urinary tract infections, wound and skin infections, bacteremia, respiratory infections, bone and joint infections, central nervous system infections, mediastinitis, pericarditis, conjunctivitis, and otitis have also been reported [3]. However, to our knowledge, this is the second case of R. ornithinolytica pyogenic non-viscerally abdominal abscess has ever been reported. Here, we describe a case of isolating R. ornithinolytica from a patient with non-viscerally abdominal abscess secondary to postoperative gastric fistula,, and the pathogenic bacteria may originate from the gastrointestinal tract.

## Case presentation

**Fig. 1:**
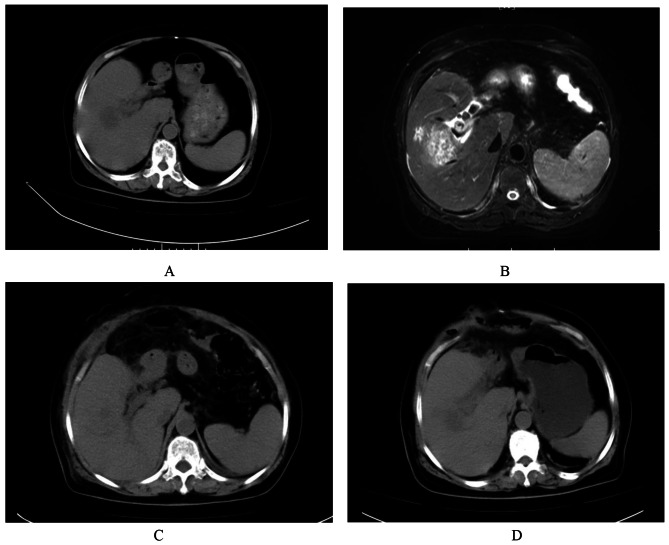
**A**) September 17th CT; **B**) NMR images showing low density lesion in the right lobe of the liver with circular enhancement, suggesting a high possibility of liver abscess and choledocholithiasis. **C**) October 1th CT scan indicates changes after common bile duct stone removal surgery; The scope of the abscess in the right lobe of the liver has decreased compared to before; **D**) October 5th CT images showing gas accumulation and exudation around the gastric antrum

A 71-year-old woman with a history of left lateral hepatectomy and cholecystectomy, presented to the emergency room with a 48 h history of fever and abdominal pain. On physical examination, the patient’s blood pressure was 131/75 mmHg, the pulse was 91 beats perminute, the temperature was 36.3℃, the respiratory rate was 22 breaths per minute. The laboratory data obtained on admission revealed a white blood cell count of 12,680/µL with 88.7% neutrophils and a C-reactive protein level of 224.5 mg/dL. Abdominal CT and MRI scan of the abdomen showed low density lesion in the right lobe of the liver with circular enhancement, suggesting a high possibility of liver abscess and choledocholithiasis (Fig. 1). Because the liver abscess without liquefaction, antibiotics treatment was considered as the main treatment option. The patient was started on treatment with piperacillin/tazobactam at a dose of 4.5 g administered every eight hours under a diagnosis of bacterial cholangitis and liver abscess.

**Fig. 2:**
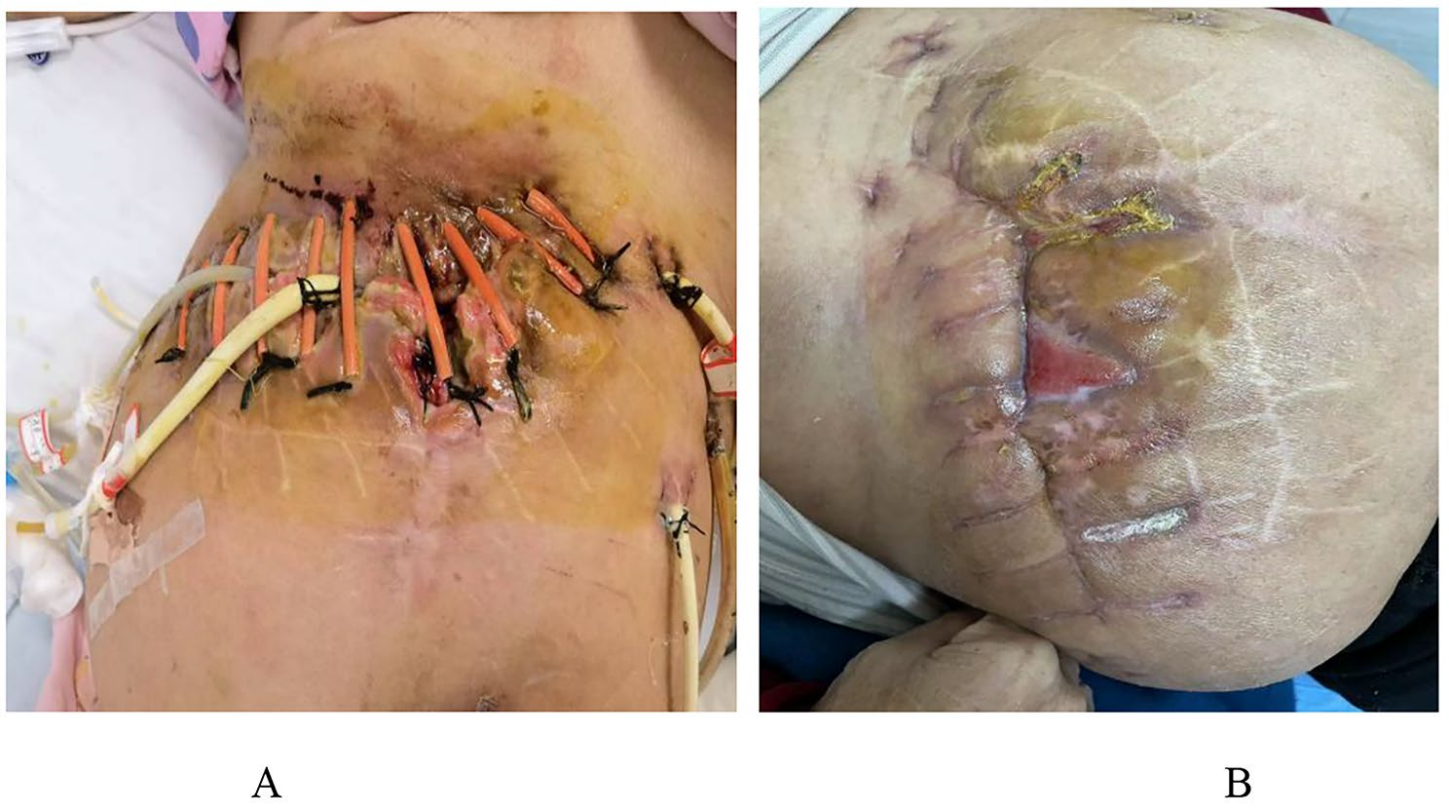
**A**) Pictures of the patient's wound on October 5th; **B**) Pictures of the patient's wound on October 23th

The treatment principle for hepatolithiasis is that removal of lesions, relief of obstruction, alleviation of stricture, and retaining of drainage of bile duct. Therefore, for the treatment of this patient’s choledocholithiasis, surgeon considered that surgery was still the best choice. On day 9, the patient’s vital indication were basically normal, and laparoscopic choledocholithotomy is planned to be performed. During intraoperative exploration, surgeon discovered extensive adhesions between the liver, stomach, duodenum, and abdominal wall. A portion of the gastric wall tissue was removed to separate the adhesions. However, Due to extensive adhesion between the common bile duct and the greater omentum, making it difficult to dissociate under laparoscopy, changed to open choledocholithotomy was performed. After the surgery, the patient clinically improved, with resolution of her fever and abdominal pain, the patient received treatment such as antibiotics and fluid infusion. On day 18, the patient experienced abdominal pain, high fever, and chills again (with body temperature fluctuating between 38.6 and 39.5 ℃). Surgeon explored the patient’s wound and found pus and food residue flowing out (Fig. 2). The laboratory data revealed a white blood cell count of 9200/µL with 83.7% neutrophils and a C-reactive protein level of 178.36 mg/dL. The patient immediately underwent abdominal CT, gastroscopy, and oral methylene blue experiment. Abdominal CT images showed gas accumulation and exudation around the gastric antrum. Oral methylene blue experiment found that blue staining liquid flows out of the wound. Gastroscopy showed duodenal bulb ulcer with residual suture and indistinct descending passage. All examination results indicated postoperative gastric leakage, then, urgent second surgery. During the operation, approximately 100 ml of purulent fluid around the liver in the upper right abdomen, and an abscess cavity measuring 4 *4 * 5 cm was observed between the left liver and stomach, containing approximately 30 ml of yellowish-white pus. A fingertip sized rupture was also detected on the posterior wall of the gastric meatus and a drainage tube was placed next to the gastric leak, which drained milky-white purulent fluid that grew R. ornithinolytica.

The R. ornithinolytica isolate exhibited susceptibility to amoxicillin/clavulanate, piperacillin/tazobactam, cefoperazone/sulbactame, imipenem, ertapenem, amikacin, levofloxacin, tigecycline and trimethoprim/sulfamethoxazole. Based on the poor overall condition of the patient, we chose meropenem to enhance anti-infection therapy. But, after using meropenem for 3 days, the patient’s condition showed no significant improvement. We added levofloxacin and after 5 days of combined treatment, the patient’s body temperature returned to normal, abdominal signs improved, and infection index significantly decreased compared to before.

## Discussion

We report the case of an 71-year-old woman with a history of choledocholithotomy presenting with fever and abdominal pain, secondary to R. ornithinolytica intra-abdominal abscess presumably due to postoperative gastric leakage. R. ornithinolytica is known as an environmental pathogen and rarely causes infections in humans but has been recognized as an increasingly important pathogen in recent years. So far, only a few of cases have been reported of abdominal infection infection caused by R. ornithinolytica, with biliary tract infection being the most common (Table 1). In 2012, Hadano et al. reported three cases of R. ornithinolytica bacteremia associated with biliary tract infections in cancer patients, all of the patients presented with fever and shaking chills with elevated hepatobiliary enzymes and jaundice [4]. In 2015, Haruki et al. summarized clinical characteristics of 11 cases R. ornithinolytica bacteremia, in which the pathogen mainly originated from the biliary tract, and found that the most common infectious focus was biliary infection and elderly patients with a history of any biliary intervention or malignancy were considered to be at a great risk for the infection [5]. In the same year, Chun et al. reported that out of 7 patients with biliary tract infections, 6 had a history of malignant tumors [6]. In 2016, Seng et al. recently reported a case series and literature review identifying 112 reported cases of R. ornithinolytica. Urinary tract infections, gastrointestinal infections, wound and skin infections, and bacteraemia were observed in 36%, 14%, 13%, and 5% of cases, respectively. Interestingly, more than half of the 16 reported cases of gastrointestinal infections were biliary tract infections. Risk factors for R. ornithinolytica infections are invasive procedures (urinary catheters, mechanical ventilation, central venous catheters), cancer, immunodeficiency, diabetes, alcoholism, and chronic kidney disease (CKD) [7]. In 2022, Alexander et al. described a case of cholecystitis and gallbladder perforation directly attributed to this species, and highlights the pathogen’s capacity to cause severe disease [8]. These cases all suggested an association between R. ornithinolytica infection and potential biliary diseases, including malignant tumors involving the biliary and/or pancreatic systems. Compared to biliary tract infections, other types of abdominal infections caused by R. ornithinolytica are relatively rare.

**Table 1:** Literature review involving R. ornithinolytica abdominal infection.

Author (reference)	Year	Clinical symptom	Age (y)/ gender	Clinical diagnosis	Treatment	Outcome
Mau et al.	2010	red skin flushing, fever	3d/M	NEC peritonitis	AMK + MEPM	Recovered
Hadano et al.	2012	Fever, chills, disturbance of consciousness,	92/M	Cholangitis	PIPC/TAZ	Recovered
		Fever, chills, disturbance of consciousness,	52/F	Cholangitis, liver abscess	CMZ	Recovered
		Fever, chills, abdominal pain.	59/M	Cholangitis	CMZ→ AMPC/CVA	Recovered
Haruki et al.	2014	-	73/F	Cholangitis	PIPC→ CAZ	Recovered
		-	75/M	Cholangitis	CFPM + AMK	Recovered
		-	92/F	Cholangitis	CPZ/SBT→ CPFX	Recovered
		-	44/M	Cholangitis	CPZ/SBT→AMPC/CVA	Recovered
		-	77/F	Cholangitis	PIPC/TAZ →CEZ	Recovered
Sibanda	2014	Fever, vomiting, abdominal pain.	53/M	Peritonitis	AMPC/CVA	Recovered
Bhatt et al.	2015	red skin flushing, fever	75/M	Sub-hepatic space infection	-	-
Hajjar et al.	2018	abdominal pain, vomiting, flushing, shock	54/M	Appendicitis	CPFX + MNZ→AMPC/CVA	Recovered
Surani et al.	2020	chronic cough	84/M	Liver abscess	CPFX	Recovered
Goggins et al.	2022	Fever, nausea, vomiting	70/M	Cholecystitis	PIPC/TAZ →ETP	Recovered

To our knowledge, only four cases have been reported so far (peritonitis, subhepatic space infection, appendicitis, liver abscess) [9–12]. Two cases had red skin flushing related to a histamine reaction. One case of patient with liver abscess, presented a chronic and non-resolving cough.

The *Raoultella spp* consists of four species: Raoultella ornithinolytica, Raoultella planticola, Raoultella terrigena, and Raoultella electrica. The *Raoultella spp* had caused a total of 4 cases of abdominal abscesses (one case of R. ornithinolytica, tree cases of R. planticola) (Table 2) [13–15]. These cases are all visceral abscesses, of which 2 patients died. The first R. ornithinolytica systemic primary purulent peritonitis was reported, and speculated that the pathogen originated from the colon in 2014 by Sibanda et al. Our case reported that separated this pathogenic bacteria from a non-viscerally abdominal abscess [16].

**Table 2:** Abdominal abscess caused by *Raoultella Spp*

Author (reference)	Pathogenic bacteria	Age (y)/ gender	Clinical diagnosis	Clinical symptom	Treatment	Outcome
Surani et al.	R. ornithinolytica	84/M	liver abscess	chronic cough	CPFX	Recovered
Sitaula et al.	R. planticola	62/M	liver abscess	abdominal pain, vomiting	CPFX	Recovered
Camposa et al.	R. planticola	52/M	pancreatic abscess	abdominal pain, vomiting	--	Die
Erwesa et al.	R. planticola	73/M	liver abscess	decreased appetite	CRO + MNZ	Die

Our patient with liver abscess, choledocholithiasis and cholangitis, and has recently undergone surgery. Therefore, she is a high-risk population for infection with R. ornithinolytica. In the present case, R. ornithinolytica was detected in the abdominal drainage fluid, but the focus of bacterial entry was unknown. Clinical doctors and pharmacists have discussed the infection pathways of pathogenic bacteria and considered that were several possible ways. (1) As a colonizing bacterium in the gastrointestinal tract, R. ornithinolytica may enters the abdominal cavity from the gastrointestinal tract due to postoperative gastric leakage. (2) After the patient’s choledocholithotomy, pathogens originating from the primary lesion of the bile duct or liver abscess undergo translocation, leading to secondary abdominal infection was a possibility. (3) Surgical procedures, invasive procedures (such as urinary catheters, drainage tubes, mechanical ventilation, and nasogastric nutrition tubes), may cause patients to acquire infections from the external environment. After the first surgery, R. ornithinolytica were not isolated from the the patient’s bile drainage fluid. However, after the second surgery, when simultaneously culturing the patient’s abdominal drainage fluid and bile, R. ornithinolytica were only isolated from the abdominal drainage fluid. The patient’s bile T-tube drainage was unobstructed which also suggested a lower possibility of secondary to biliary tract infection. From the first day of hospitalization to the second surgery, triple abdominal CT scans of the patient all showed further reduction of the liver abscess, indicating that medical treatment for liver abscess was effective. The main source of pathogenic bacteria in liver abscess is through biliary and blood dissemination. This patient’s bile culture is negative, and considering all factors, the possibility of secondary liver abscess was relatively low.

However, clinical symptoms such as abdominal pain, high fever, and chills all recurrent occurred after gastric fistula. The appearance of clinical symptoms and the rebound of infection indicators confirmed that abdominal abscess was secondary to gastric leakage. Previous studies have reported that the translocation of R. ornithinolytica colonizing the gastrointestinal tract leads to infection [17]. For the above possibilities, we considered that pathogenic bacteria were more likely to originate from the gastrointestinal tract.

Due to R. ornithinolytica production beta-lactamases that makes this agent naturally resistant to ampicillin [18]. Research reports indicate that R. ornithinolytica are sensitive to most antibiotics, including cephalosporin third-generation, fluoroquinolones, carbapenems, and aminoglycosides. In our case, R. ornithinolytica are resistant to ceftriaxone and ceftazidime, but sensitive to cefoperazone sulbactam and also show sensitivity to fluoroquinolones, carbapenems, and aminoglycosides. Although infection caused by R. ornithinolytica is relatively rare in clinical, it causes serious and often life-threatening infections and high drug-resistance rate are worth noting. Chun et al. reported that out of 16 patients with bacteremia, 7 patients died due to infection [6]. Etani et al. found 6 patients with R. ornithinolytica bacteremia, of which 2 patients died with accompanying cholecystitis [19]. Li et al. and Abayomid et al. both isolated multidrug-resistant R. ornithinolytica and ultimately led to the patient’s death [20]. Although favorable antimicrobial susceptibility test results, in patients with malignancies or weakened immunity, infection with R. ornithinolytica often leads to life-threatening infections such as sepsis and multiple organ failure. With the increase of drug-resistant bacteria in the environment and the transfer of bacterial drug-resistant genes, it poses challenges for nosocomial infections [21].

## Conclusion

R.ornithinolytica is a virulent pathogen causing community-acquired and hospital-acquired infection, especially in immunocompromised populations. In the present report, R. ornithinolytica was the etiologic agent responsible for abdominal abscess. The patient was successfully treated with surgical drainage and a 10-daycourse of antibiotics, including meropenem and levofloxacin. This case reported a non-viscerally abdominal abscess caused by R. ornithinolytica secondary to postoperative gastric fistula. This case adds to the spectrum of disease caused by R. ornithinolytica and illustrates invasive medical procedures can be major risk factors for active infection due to this organism. Therefore, it is prudent to consider the possibility of R. ornithinolytica as a causative agent among those with the above risk factors.

## Data Availability

The data used to support the findings of this study are included within the article.

## Abbreviations

MNZ: metronidazol
CPFX: ciprofloxacin
CRO: ceftriaxone
AMPC/CVA: amoxicillin/ clavulanate
AMK: amikacin
CFPM: cefepime
PIPC/TAZ: piperacillin/tazobactam
CMZ: cefmetazole
CPZ/SBT: cefoperazone/sulbactam
ETP: ertapenem
